# How can technology support quality improvement? Lessons learned from the adoption of an analytics tool for advanced performance measurement in a hospital unit

**DOI:** 10.1186/s12913-020-05622-7

**Published:** 2020-09-01

**Authors:** Sara Tolf, Johan Mesterton, Daniel Söderberg, Isis Amer-Wåhlin, Pamela Mazzocato

**Affiliations:** 1 Department of Learning, Informatics, Management and Ethics, Medical Management Centre, Karolinska Institutet, Stockholm, Sweden; 2 Ivbar Institute, Stockholm, Sweden; 3 Department of Women and Child Health, Karolinska Institutet, Stockholm, Sweden; 4 Department for Research, Development, Education and Innovation, Södertälje Hospital, Södertälje, Sweden

**Keywords:** Obstetrics, Quality improvement, Technology, Complexity, Performance measurement

## Abstract

**Background:**

Technology for timely feedback of data has the potential to support quality improvement (QI) in health care. However, such technology may pose difficulties stemming from the complex interaction with the setting in which it is implemented. To enable professionals to use data in QI there is a need to better understand of how to handle this complexity. This study aims to explore factors that influence the adoption of a technology-supported QI programme in an obstetric unit through a complexity informed framework.

**Methods:**

This qualitative study, based on focus group interviews, was conducted at a Swedish university hospital’s obstetric unit, which used an analytics tool for advanced performance measurement that gave timely and case mix adjusted feedback of performance data to support QI. Data was collected through three focus group interviews conducted with 16 managers and staff. The Nonadoption, Abandonment, Scale-up, Spread, and Sustainability (NASSS) framework guided the data collection and analysis.

**Results:**

Staff and managers deemed the technology to effectively support ongoing QI efforts by providing timely access to reliable data. The value of the technology was associated with a clear need to make better use of existing data in QI. The data and the methodology in the analytics tool reflected the complexity of the clinical conditions treated but was presented through an interface that was easy to access and user friendly. However, prior understanding of statistics was helpful to be able to fully grasp the presented data. The tool was adapted to the needs and the organizational conditions of the local setting through a collaborative approach between the technology supplier and the adopters.

**Conclusions:**

Technology has the potential to enable systematic QI through motivating professionals by providing timely and adequate feedback of performance. The adoption of such technology is complex and requires openness for gradual learning and improvement.

## Background

Quality Improvement (QI) in health care has been described as the combined and continuous actions that lead to better patient outcomes, better system performance, and better professional development [1]. For QI to be effective in health care, performance measurement is required to provide feedback to professionals and organizations on the quality of care provided [2]. Feedback on performance may increase health care professionals’ and managers’ learning in ways that can result in changes in practice that can be retained, modified, or rejected [1–4]. However, despite many health care organizations engaging in feedback and QI, few manage to consistently improve quality and sustain results over time [4, 5].

In part, this may be explained by the challenges involved in providing relevant feedback to professionals. Analyses of interventions involving audit and feedback show mixed results, with a number of different factors related to the type of feedback impacting its effectiveness [6]. One hindrance links to the requirement that performance data must be fed back continuously and in a timely manner [7, 8]. A systematic review reveals that the QI tool referred to as the Plan-Do-Study-Act (PDSA) is often used with no access to data at weekly or even monthly intervals [4]. Research even shows that time lags in data feedback can be as long as 3 years [8]. Consequently, health care professionals and managers are unable to continuously evaluate the changes [4]. Lack of trust in underlying data also affects the use of feedback and studies show that case mix adjustment, i.e. adjustment of data for differences in patient characteristics, is important factor for trust among professionals [7, 9].

Improvements in technology have advanced the ability to capture, process, analyze, and present data [10]. While performance measurement used to be a largely manual process, technical solutions can now incorporate data from different databases (e.g., claims data, quality registers, and electronic health records [EMRs]), adjust for differences in patient characteristics, and quickly analyze data.

However, despite the potential for technology to support QI, implementation of such programs is often challenging and may often result in less than satisfactory results [11, 12]. A wide body of evidence indicates that several attributes of a technology itself, such as its potential benefits, user-friendliness, compatibility with organizational values and its complexity, influence the innovation’s adoption into health care organizations [13, 14]. During the last decades, increasing academic attention has also been given to contextual factors, such as adopters, organizational aspects and leadership as well as political and economic influences that affect the adoption of technologies [11–16]. Greenhalgh et al. suggest factors influencing the adoption of innovations in health care can be categorized into seven different domains pertaining to the technology itself as well as system into which it is being introduced [13, 17]. Furthermore, their research shows it is not individual factors themselves, but rather the dynamic interaction between them, that determine the adoption of technological innovations in health care. Health care organizations tend to underestimate this complexity [17]. Technologies tend to be over-simplified, poorly prototyped, and inappropriately customized, which can result in early rejection and abandonment [12]. A deeper understanding of the dynamic interaction between the technology and the context in which it is implemented can guide successful adoption [13, 18].

Thus, this study aims to explore factors that influence the adoption of a technology-supported QI programme in a hospital unit, through a complexity informed framework.

## Methods

This qualitative study, based on focus group interviews, was conducted at the obstetric unit of an obstetrics and gynecology (OB/GYN) department at a Swedish university hospital. We selected the unit because of its work with an innovative and technology-supported QI programme.

### Theoretical framework

This study explores the adoption of a technology for advanced performance management to support a QI programme. The study was guided by a theoretical framework that was specifically developed to understand how complexity influences the adoption of technology-supported programmes, i.e. the Nonadoption, Abandonment, Scale-up, Spread, and Sustainability (NASSS) framework [13].

The framework has been previously empirically used to explain the success or failure of technology adoption in health care [13, 19]. Seven domains (D) are included in the framework: clinical condition, technology, value proposition, adopter system, organization, wider system, and embediness and adoption over time (Table 1). Building on complexity theory [18, 20], the framework suggests that each domain can be classified as simple (“straightforward, predictable, few components”), complicated (“multiple interacting components or issues”), and complex (“dynamic, unpredictable, not easily disaggregated into constituent components”). The framework helps to understand how the inherent complexity ant the interactions between the domains can influence the success or failure of technology supported programme [13]. The more complex technology supported programs are the more these interactions can be expected to be constantly changing, unpredictable, and non-linear [18, 21].

**Table 1:** Description of the seven domains in the NASSS framework

Domain (D)	Description
Condition (D1)	The nature or characteristics of the condition or diagnoses that the technological innovation address, as well as relevant co-morbidities and sociocultural aspects.
Technology (D2)	The technological features of the innovation, such as its design and perceived usability, the quality and reliability of knowledge generated as well as the skill and support needed to use the technology. It also concerns the long-term sustainability of the technology, such as possibility of adaptations and potential market dynamics that may impact the future availability of the product.
Value proposition (D3)	The expected value of the technological innovation, both from a supply-side business model view, and from the perspective of the health provider, weighing potential benefits for patients against costs of procurement.
Adopter system (D4)	Changes in staff roles or responsibilities that threat professional identities are factors that add complexity and may impede implementation of new innovations. The domain also includes expectations on patients’ or their caregivers’ knowledge and involvement in innovation adoption.
Organization (D5)	The organisation’s readiness to adopt new technology, how the decision to implement the technology into the organisation was made and how that decision was motivated and funded. Disruptions to established work routines and the amount of work required to adopt the new technology may as well affect organisational response.
Wider system (D6)	Political, financial, regulatory/legal and social context that may influence the means and successfulness of the technology into the organisation.
Embedding and adaptation over time (D7)	The possibility to “coevolve” technology to changing context within the organisation and the resilience of the organisation in adapting to unforeseen events, which can impact the ability of the organisation to retain and further develop technology over time.

### Setting

The obstetric unit in the OB/GYN department provides birth care for 4.200–4.300 women annually as well as related pathology and recovery care. The university hospital in which the unit is located has beyond its regional catchment area also referrals from northern Sweden for fetal medicine.

In 2013, the Swedish government approved financing for a national, cross-regional research and development project called Sveus, which provided the foundation for the clinic’s QI programme. Sveus aimed to develop methodologies for continuous measurement of case mix adjusted performance. This research resulted in the launch of a cross-regional analytics tool for case mix adjusted benchmarking of outcomes, resource use, and care processes. Birth care was one of the six patient groups initially addressed in the project [22–24]. Moreover the government decided to put improvement of birth care and women’s health on the political agenda and pledged significant national funds to the regions between 2015 and 2022 [25].

### The technology supported QI programme

In 2017 the obstetric unit launched a technology-supported QI programme as part of a hospital wide Value Based Health Care (VBHC) effort that involved the use of an analytics tool, Era (Ivbar Institute AB, Stockholm, Sweden), for advanced performance measurement. The unit paid a license fee for use of the tool. The tool included data from several sources, one of which is the cross-regional benchmarking tool used by the regions participating in Sveus. This data was primarily based on information from patient administrative systems and included algorithms for case mix adjustment [23]. This data was combined with clinical data from local EMRs to enable tracking of local performance on a wide array of different indicators. Different dashboards for performance measurement were available to the clinic through web-interfaces and were all updated weekly to ensure timely feedback of performance. The dashboards included:

1. An overview dashboard for managers group with granular information on volumes, indicators of care process, resource use, outcomes and patient satisfaction. The dashboard included information on performance in different subgroups of patients, tracked ongoing improvement projects, and presented information on indicators where the clinic performed better and worse than expected based on information about case mix-adjusted performance from the cross-regional tool.
2. Dashboards available for managers and staff actively engaged in the QI programme with detailed information of development of indicators related to ongoing improvement projects, including analysis of different subgroups of patients.
3. A dashboard made available to the entire staff with information about selected important performance indicators, a list of indicators where the clinic performed better/worse than expected, as well as information on ongoing improvement projects.

The adoption of the tool in 2017 led to the launch of five improvement initiatives organized into multi-disciplinary QI teams that consisted of physicians, midwives, and assistant nurses. These teams focused on reductions in the rates of caesarean deliveries, labour inductions, post-partum infections, newborns with low Apgar score and urinary retention. The choice of improvement initiatives was largely based on identified improvement potential from the cross-regional benchmarking performed within Sveus.

### Data collection

Data were collected in three focus group interviews (16 informants, with 4–6 participants in each group) in September and October of 2018. We chose to conduct focus group interviews because QI at the unit was conducted in multi-professional teams, and thus we wanted to promote group discussions around the potential benefits of using technology to support QI [26]. A semi-structured interview guide with open-ended questions (Additional file 1) was used that addressed the seven domains in the NASSS framework (Table 1), and thus included questions concerning: D1) what characterizes the patient group treated in the unit; D2) how the tool was used to support QI; D3) the perceived value of the tool; D4) changes in the adoption system needed for the use of the tool; D5) organizational aspects related to the adoption; D6) aspects of the wider system that influenced the adoption; D7) and the embeddedness and the adoption over time. The Consolidated Criteria for Reporting Qualitative Research (COREQ), which is a 32-item checklist, was used to enhance the reporting of the findings [27].

The multidisciplinary research team consisted of: a health economist (JM), two physicians (IAW, DS), a sociologist (ST), and senior researcher in medical management (PM). Collectively, the team has experience in the theory and practice of QI and organizational change (PM, IAW, ST), obstetrics (IAW), performance measurement (IAW, JM), health economics (JM), and medical management (PM, IAW, ST).

Purposive sampling was used to select informants who could provide a rich and diverse perspective on the potential benefits and challenges of technology-supported QI [28, 29]. Based on this we included managers, staff actively engaged in the QI programme, and staff not actively engaged in the QI programme. The latter were included because we expected them to have had experienced the technology-supported QI programme even if they were not directly involved. The diversity among the informants aimed to provide varied experiences and perceptions [26].

The head of the obstetric unit was tasked with identifying possible informants who met the selection criteria, as she had the main responsibility for the QI programme and hence knew which personnel was involved in the programme or not. She recruited informants by speaking with them directly or emailing them, there were no record of informant drop out in the recruitment process. The participants were grouped into three groups: managers, staff involved in QI and staff not involved in QI. Informants included are summarized in Table 2.

**Table 2:** Participants of focus groups

Group:	Staff not involved	Staff involved	Managers	Total
Physicians	2	2	2	6
Midwifes	2	2	2	6
Assistant Nurses	2	2	0	4
Total	6	6	4	16

The interviews lasted from 75 to 90 min each and were audio-recorded and transcribed. A facilitator (PM or ST) led the interviews. One or two researchers (DS and/or ST) observed the interviews. Before the interview participants were also verbally informed about the educational background and field of interest of the interviewers. Interviews were conducted at the participants’ workplace.

### Data analysis

We analyzed the data through directed content analysis, i.e. a deductive approach to analysis [30, 31]. We chose a deductive approach because the NASSS framework had previously identified key domains important to consider in the adoption of technology-driven programmes. Therefore, we developed an a priori code book based on the definition of the domains in the NASSS framework [13]. The content analysis process followed seven steps. First, two researchers (ST and DS) read the transcribed interviews to get a sense of the material. Second, these two researchers condensed the data into condensed meaning units, i.e. reduced meaning units into shorter text. One interview was condensed by both researchers independently and then compared their results to ensure consistency in level of condensation. Thereafter DS condensed the text in the second interview into condensed meaning units and ST the third interview. Third, condensed meaning units where printed and placed randomly on a table; ST, DS and JM sorted the condensed meaning units independently and in silence into the a-priori defined categories (D1-D7) based on the NASSS framework and developed subcategories. The subcategories were identified by grouping condensed meaning units with related meanings. Fourth, all authors revised the sorting of the condensed meaning units into the a-priori defined categories and together further developed the subcategories informed by negotiated consensus [32]. Fifth, the authors developed synthesized descriptions of the empirical databased on the subcategories the authors. Sixth, all authors read through the descriptions of each domain and independently categorized the domains into simple, complicated, or complex. All authors articulated their reasoning and discrepancies were identified, discussed, and resolved. Seventh, validation with participants from the focus groups as well as other employees was performed by PM and ST by presenting the results in a workshop. All participants in the validation session were asked to independently mark the emerged categories within the a priori domains with agree or disagree. Informants confirmed that the findings mirrored their experience. Microsoft Word and Nvivo 12.0 were used to manage the data. The datasets generated and/or analysed in this study are not publicly available to maintain confidentially, but de-identified data are available from the corresponding author on reasonable request.

## Results

The results section includes first a presentation of the empirical findings, based on the subcategories that were identified (Table 3) and linked to each of the seven domains in the NASSS framework, followed by an analysis of how the complexity inherent in each domain and the interaction between the domains influenced the QI programme.

**Table 3:** Description of subcategories linked to each domain of the NASSS framework

**Domain**	**Subcategory**
Condition (D1)	Broad patient population including both high and low risk patients with diverse background.
	Large birth clinic also accepting patients from other regions.
Technology (D2)	The platform enabled staff to easily understand data and to gain new knowledge; prior understanding of statistics was helpful.
	The tool was easily accessible.
	Timely feedback of data made it more relevant for QI than historical data previously used.
	Case mix adjustment made the data more relevant for QI.
	Highly detailed data was important for its use in QI.
	Lack of detail in certain data in the tool resulted in the need of additional data collection from other sources.
	Adaptation of the platform to meet future needs was deemed possible.
	Data was generally considered reliable and credible. However, use of data led to increased awareness of differences in measurement for certain variables which led to discussion about data quality and coding.
	Collaboration and a mutual learning process with the platform supplier enabled the continuous adaptation of the platform tool to the clinic’s specific needs.
Value proposition (D3)	The cross-regional Sveus report created an awareness of around differences in performance and the possibility of using data for QI purposes.
	Previously used data was not suitable for QI due to large time lag and poor accessibility.
Adopter system (D4)	The clinic had an established culture of working interprofessionally.
	Interprofessional teams were thought to create unity around improvements and lead to better care for patients.
	Increased use of measurable data could create excessive focus on risk groups over “normal” patient groups, groups over the individual patient, and measurable aspects over unmeasurable aspects of care.
	The project did not require any significant changes in staff roles but led to the need of development of some new skills in the clinical work.
	There was an ambition of evolving staff’s role in using data independently, but lack of hindered this development.
	Data visualized the need for change and improvements in results and triggered a dialogue which motivated staff to further improve.
	Data united staff around a common understanding of the current situation and emphasized the need for change.
	Better understanding of data could improve information to patients.
Organization (D5)	Data was not available for staff who were not involved in QI.
	Staff not involved in QI had little insight into the innovation and the work done within QI teams.
	Staff not involved in QI were involved only as recipients of directives decided within QI teams.
	Even though the ambition was that anyone should access the data, in practice the managers and staff involved in QI were the ones who used it and then presented it to the staff.
	An already established digital way of working facilitated the use of the tool.
	A clear mandate was perceived to be needed to conduct QI.
	A long learning process was required for managers to understand the tool before beginning its implementation in the clinic.
	Use of the tool was promoted by managers through creating a curiosity and demand for the information, which was intended to secure longevity of the initiative
	Engaged manager enabled the implementation of the project.
	Recruitment to QI teams was based on expressed interest or decided by managers.
	Lack of time and difficulty scheduling limited staff’s ability to work with QI and use the data.
	Use of data led to improvements in data registration in order to improve data quality.
Wider System (D6)	Data improved communication around patients with other sections of the women’s clinic, the neonatal clinic and external actors.
	Medial discussion about the quality of birth care caused concern amongst patients
	The Region had a goal to reduce infections, but this was not important for the initiation of the QI programme.
	A hospital-wide programme on VBHC supported the implementation of the tool to some extent.

### Condition: pregnancy spans from simple to complex (D1)

Representatives from all focus groups described that the unit treated a broad patient population e.g. both emergency and elective care took place at the unit and that some patients were low-risk in normal labour while others were high-risk with complex conditions such as premature delivery and maternal co-morbidity.

### Technology: a practical but not trivial analytics tool (D2)

Representatives of the managers and staff involved in QI described that the feature of case mix adjustment made data more relevant compared to unadjusted data and counteracted the practice of justifying poor performance outcomes with misconceptions about patient complexity.

*(One informant) – And the case mix adjustment has made a difference. Before we blamed a lot on the fact that our patients are so special. (Another informant) – Yes, absolutely, [we said] “We have so difficult patients” and “It’s a little bit special here”.* [Managers]

Staff involved in QI teams expressed that the level of data detail was generally high which was considered important for its usage, although in some cases it was too coarse. Moreover, both managers and staff involved in QI perceived the timelines of data feedback to be relevant and useful.

*(One informant) - Now it is more easily accessible. (Another informant) - Quickly look at recent data that are divided into different focus areas so that you can quickly get an overview as you say. “The last month something has happened, it is suddenly 30% caesarean sections, what should we do?”* [Staff involved in QI teams]

The managers said the dashboards were easily available on any devices such as computers, smart phones, and tablets. The graphic presentation of the data via the web interface was perceived as understandable, user-friendly, and clear by managers and staff involved in QI-teams. The staff who were involved in QI teams reflected on their different preferences concerning the visual presentation of data and they suggested that further improvements in the interface would increase data accessibility even more. The two staff groups said more guidance and support was needed for identifying, selecting, extracting, and understanding relevant data. It was also described by involved staff that prior understanding of statistics was helpful to be able to fully grasp the presented data.

Representatives from the manager group and staff involved in QI said that the relationship with the supplier was such that it was possible to customize the analytics tool to the local needs and conditions. For example, in addition to indicators established through the cross-regional benchmarking, the supplier facilitated measurement of indicators specifically demanded by the unit, incorporated local data available in the department’s EMR, and adapted to the hospital’s system for data transfer. Involved staff described how they contributed with their clinical knowledge to identify what data was needed to guide QI. The supplier was in turn able to translate these needs into data demands to the hospital-IT department.

### Value proposition: timely and reliable data (D3)

According to informants from all focus groups, the analytics tool was needed because of the existing QI challenges. The managers described that a previous cross-regional report, from the Sveus project, suggested there was room for improvement in a number of areas. This report was said to motivate the unit to require more data to better understand performance and to initiate improvement activities. It became clear that there were areas of underperformance in the unit, and that the patient mix was not the cause of the variations. The managers described that even before the introduction of the technology-supported QI programme, data were seen as essential to QI in the unit. However, prior to the adoption of the technology innovation data were often difficult to access and were often out-of-date.

*(One informant)- Statistics has been our weak spot. We’ve been able to measure but it has been difficult. And really difficult sometimes when we wondered:* “*How many women with diabetes do we have?” or* “*How many complications do we have?”. So it was very difficult to get that data (Another informant) – It was basic, with a pencil and put into Excel-files.* [Managers]

### Adoption system: managers and QI teams use the tool for improvement purposes but need complementary data to capture the patient perspective more comprehensively (D4)

The managers and the staff involved in QI teams were the main adopters of the analytics tool. They described how they used the tool to identify performance deviation and improvement needs that were grounded in valid information. They also used the tool to evaluate the effect of changes to the unit’s protocols.

Despite the clear use of the tool for improvement purposes, the adopters reflected on the need to integrate other sources such as surveys, focus groups, and individual interviews, to complement the data provided by the tool with data on patient perspective. Medical records were also reviewed. Taken as a whole, all these data sources were used to make changes to the unit’s protocols. In some cases, new protocols were adopted that required the staff to acquire new skills and to develop new competences.

Some informants reflected on the potential risks of using performance data in QI. The managers worried that the improvement efforts would shift focus to certain metrics and to aggregated patient groups over the needs of the individual patient. Staff were concerned that the improvement efforts would focus on measurable areas to the exclusion of areas that were not easily quantified.

According to the managers, one goal of the QI programme was that all managers and staff should be able to easily access and use the analytics tool. In practice, staff not involved in QI had limited or no knowledge of, or experience with, the tool; yet they expressed an interest in learning about the improvement efforts as they talked about the tool.

*(One informant) - I haven’t even seen it. (Another informant) - Nor have I. (Yet another informant)- I think we should see it more. (The first informant)- You could do it very shortly in a group like this. One afternoon, just bring up that:* *“This is what it looked like 3 months ago and today it looks like this. Look how good it is”.* [Staff not involved in QI teams]

This was further corroborated by the managers who said it would be beneficial if staff could start to use the tool independently; however, this would require time and knowledge that was not available.

One manager said she could use the data to communicate with patients about the medical risks associated with their conditions and staff members said that data helped them to feel more confident when they explained the reasons behind their decisions to patients.

*“And then be able to present to the patients also that*” *If we induce labour in this way then we have a high proportion who deliver vaginally, where everything works well, and these are the risks if you start to induce labour very early. So that you also have a solid fact base for your own sake.* [Staff involved in QI teams]

### Organization: the tool supports multidisciplinary QI-efforts and emphasizes the need for change (D5)

Several organizational factors were described to have supported the adoption of the analytics tool such as the multidisciplinary approach to QI, formal education seminars and workshops, and an implementation approach that focus on demonstrating the benefit of the tools.

Managers saw the unit as a pioneer in QI as they had run several QI initiatives, such as lean. The unit had used data in QI efforts before and had a practice of working in multidisciplinary teams. Staff involved in QI described how the improvement work done in multidisciplinary teams did not use a specific and standard approach to QI. Instead, the teams self-determined how to organize their work. Participants in QI teams were included on a voluntary basis or via appointment by managers. However, representatives from all three groups expressed that an impediment to engaging in the QI teams was insufficient time and that meeting times conflicted with clinical engagements.

The managers described that the long experience of working in multi-disciplinary teams supported the implementation of new clinical routines owing to the diversity of knowledge of, and experience with, current clinical practices and created unity around changes.

The managers also described that formal education seminars and workshops also promoted acceptance of the QI programme. However, the staff who were not involved in the QI programme described themselves as “passive” recipients of the new practices. It was not clear to them how the new routines were developed or by whom. The staff who were engaged in the QI programme acknowledged the need for better communication and interactions between the QI teams and other employees.

Staff involved in QI teams described that the head of unit played an important role in the adoption of the analytics tool since she had great interest in development of care and increased patient safety. The managers described that her approach to implementing the tool was to demonstrate the possibilities the tool offered. The managers argued that this was a strategy to ensure the sustainable adoption of the tool, and the technology-supported QI was seen as a long-term ambition as much as an immediate goal.

Both involved staff and managers described that the improved access to data unified staff and managers around their interpretation of current performance and the goals of the QI programme. The managers described how they used the data to motivate staff by calling attention to the variations between observed and expected levels of performance indicators. They also used the data to generate greater interest in performance measurement and to improve the dialogue with other hospital departments.

*But it is also the fact that it becomes easier with the communication to the staff. Because data has been old previously and what kind of feedback is that? People could say …* *“Yes, that’s the way it was then, when they worked here, and not me”. Now, we are looking at two-week old data … I believe the discussion is much more here and now which also makes it easier to motivate.* [Managers]

Both manager and involved staff mentioned that the increased focus on performance measurement and benchmarking led to an increased and shared understanding of the importance of data reliability. This gradually had led to efforts made to improve routines for data recording. For example, changes were made to standardize the data entries in the EMR system.

### Wider system: the political and societal debate reinforced the need for improvement in obstetrics care (D6)

Staff involved in QI mentioned how the ongoing societal and political debate emphasized the need to improve birth care. For example, media reports on the quality of birth care had created concern among patients which triggered the need to act upon this situation.

Some objectives of the QI programme, such as the reduction in the number of infections, were identified by involved staff as goals set at the county level. These externally set goals only indirectly affected the units efforts to improve birth care.

The managers reported that the QI initiative at the obstetric unit linked well to VBHC, which provided additional support for the QI programme.

### Embedding: close collaboration with the supplier enabled adaptation over time (D7)

The research mainly focused on the early adoption of the QI programme. Thus, only limited findings were identified concerning D7. A factor that contributed to adaptation over time was the close collaboration between the supplier of the tool, the managers, and the staff to adapt the tool to local needs and conditions. They learned that data reliability and validity were essential and could not be taken for granted.

### Analysis of the interaction between NASSS domains

Patients treated at the unit ranged from simple, i.e. low risk, to complex, i.e. high-risk patients and with varying comorbidities (D1). In this setting, the new technology provided case mix adjusted performance indicators that enabled staff and managers to better understand the complexity that characterized their patients and to trust the performance measurement (D2).

Even with the case mix adjusted data, the data provided in the tool were not always sufficient to fully grasp quality. Therefore, multiple data sources were used to complement the tool. These factors complicate the adoption since multiple components and agents need to interact to get a broad perspective on performance (D4).

The analytics tool required significant adaptation from both the supplier and its adopters. It was necessary to customize the performance indicators and to integrate the technology with existing data systems (D2). Thus, the supplier and the adopters modified and co-developed over time specific features of the tool. This complex adaptation process ultimately resulted in a simpler and more practical technology that produced timely and reliable data (D2). The high desirability of the technology (D3) also contributed to make the technology a good fit for the adopters, i.e. the possibility the technology offered to support QI based on reliable data.

The relative simplicity of the adopter system, as limited changes were needed to staff roles and routines (D4), combined with several organizational factors, reduced the uncertainty associated with the new technology. The QI programme was limited to the obstetric unit where the multi-disciplinary QI teams were already in place and were led by a motivated leader (D5). All this contributed to a successful initial adoption. However, an observation was made that staff not involved in QI were not fully aware of the technology.

As far as the wider system (D6) limited insights were gained from the interviews. The hospital-wide VBHC initiative, however, seemed beneficial since it linked to the QI programme. Our previous knowledge of this area suggests that other important factors in the wider system may have facilitated the local adoption process. The national, cross-regional benchmarking initiative, Sveus, which dealt with a number of challenges related to data collection, informatics, methodology, and legal issues, had smoothed the technology adoption path for the QI programme. Moreover, previous work by Sveus had helped to legitimize the selection and definition of relevant birth care indicators. This can be argued to have reduced the complexity of the adoption.

## Discussion

Staff and managers in the obstetric unit deemed the technology to effectively support ongoing QI efforts by providing timely access to reliable data. The adoption of the technology was facilitated by several factors. There was a clear need to make better use of existing data in QI. The underlying data and the methodology reflected the complexity of the clinical conditions treated yet was presented through an interface that was easy to access and user friendly. Moreover, the approach was adapted to its specific setting by acknowledging the importance of local needs and organizational conditions and by recognizing that a collaborative approach between the supplier and the adopters was essential. At the organizational level, the managers created the conditions that allowed the staff to engage in QI and motivated them by demonstrating the potential benefits that the technology offered. The managers and the head of unit also understood the importance of adaptation and learning over time. The QI programme was embedded in a wider social and political system which included the hospital’s promotion of VBHC and the national government initiative to improve birth care.

A key learning from this study is that technology can support QI by providing timely data feedback which is critical to engage professionals and to allow for quickly evaluating the impact of improvement activities. Before the adoption of the QI programme, the obstetric unit relied on a variety of data sources such as published reports and Excel files that were not easily accessible and were often out-of-date. The positive effects of performance measurement often do not materialize because of problems with access to data and insufficient resources for data collection [33, 34]. Time delays in follow-up undermine clinicians’ confidence in data [35, 36] and reduce the accountability for outcomes [37].

This study confirms that adjustment for case mix is important to provide more meaningful information about actual performance and to avoid that professionals focus on potential differences in their own case mix (the “my patients are sicker” syndrome) rather than on areas for improvement of clinical practice [7]. While adjustment for case mix involves complex data analysis, it reduces complexity in the interpretation of the results. The study also shows that reliability of underlying data is a prerequisite for accurate performance measurement. The systematic QI work with data in the obstetric unit revealed some problems with poor data quality. A technology-supported QI programme can be a catalyst that sparks improvements in medical and administrative data reporting and coding.

Moreover, the increased availability of data used by the multi-disciplinary teams helped to create a common view of the clinic’s performance and improvement needs. Performance measurement is a complex task and multiple stakeholders must be convinced of the relevance and motivational power of performance measurement. While health care professionals may be thought of as a homogenous stakeholder group, they often have very different views on quality of care [29]. Physicians, for example, tend to concentrate on the outcomes dimension while nurses tend to concentrate on the experience dimension [20, 38]. This study illustrates how data can be used in QI teams to create a consensus around the performance measures and help create unity around the need for improvement. The staff in this study thought the systematic performance measurement gave them more confidence in their communication with patients on medical decisions. Yet, the patients, who were not adopters or users of the new technology, had no access to it. Possibly more involvement of patients could be an area for development, for instance, by giving them additional information on expected outcomes. However, sharing complex statistical data with patients poses other challenges. It requires a familiarity both with data and with the interpretation of data and it is essential that statistical data can be explained meaningfully to patients.

The collaborative approach with the supplier of the technology was important to develop indicators relevant for the local setting. Previous research shows the need to involve professionals in performance measurements [39]. If professionals do not take ownership of quality indicators, the value of the performance measurement declines [33, 34, 40, 41]. Enhanced quality in care will not be achieved with just measurement of performance and timely and reliable feedback of data but also requires effective ways to engage those who should perform the improvement work [40].

The NASSS framework was helpful to understand how the early adoption process of a technology-supported QI programme was facilitated by a technology that was able to match the complexity of the clinical condition treated. This was in turn enabled by an adoption approach that focused on adapting the technology to the local needs and infrastructures. The adaptation process required a collaborative approach that focused on learning rather than mere implementation. The learners were the tool supplier, the managers and staff in the obstetric unit, other hospital administrators, and even the wider political and civic communities. Previous research concludes that, without learning, technologies are more easily rejected or abandoned when they are over-simplified, poorly prototyped, and inappropriately customized, with unclear value propositions [12, 33]. Complex challenges that arise when new technologies are adopted require the involvement of multiple stakeholders who can find solutions to the problems encountered [34]. Organizations must be resilient as well as focused when they adopt new innovations (e.g. technologies). The resilient organization cannot reasonably expect that the innovation will immediately function perfectly [38, 41]. Instead, the resilient organization should work collaboratively with the technology supplier as problems are solved and adaptions suited to the local context are made. Step by step, actions should be taken to integrate technology and be open for gradual improvements in the use of technology over time, both in terms of actual improvement of the technology and data itself, but also in how it is being used in the daily work.

The extensive learning and adaption phase described in this study facilitated the adoption of a complex technology. However, that process may also present a challenge when it is time for the scale-up and spread phases. Improving semantic inter-operability between different systems in health care would reduce the burden of local technology integrations and would facilitate scaling-up and spreading that technology [36]. More research is needed that explores how customization and adaptation over time may influence long term spread, sustainability and scale up of technologies aimed to support QI programmes.

### Strengths and limitations

While there are many hospitals using advanced analytics systems providing performance data to improve quality in care, few studies qualitatively investigate how these technological solutions are integrated in and impact QI efforts [42]. This study takes a wide perspective of the experiences of both leaders, staff involved in QI and staff not involved in QI [19]. However, one limitation of the study is that it focuses on the early adoption phase in one setting and it does not fully capture data concerning the wider system and the embeddedness and adaptation over time. Thus, saturation was not fully reached for the domains six and seven. Saturation in deductive studies refers to extent to which pre-determined codes or themes are adequately represented in the data [43]. The description of domains six and seven could have been enriched by including perspectives from stakeholders external to the hospital and a longitudinal perspective on the adoption of the technology.

One potential limitation is the risk of selection bias in group participants, as the head of unit, who had been a driving force behind the QI programme, recruited informants. She was very familiar with who belonged to which group and had knowledge about the schedule of staff, and we deemed it would be difficult to recruit informants in another way. This selection process may however have influenced selection of respondents. This selection bias was counterbalanced by the inclusions of staff and managers who played different roles in the QI programme. This minimized the risk for power imbalances which could have hampered interviewees to speak freely and also gave a better understanding of adoption and spread throughout the organization. Another potential limitation is the number of participants in each focus group. Nyumba et al. report that 4–15 participants is common [29]. In the focus groups in this study the number ranged from 4 to 6 and was determined by the size of the organization and the number of participants that could partake in each group without compromising the clinical work at the department. While the size of the groups may be seen as a limitation in our study it also enabled us to have homogeneous groups, in accordance with the recommendations from Nyumba et al. 2017 [29].

Finally, the analysis was strengthened by the multidisciplinary team of researchers, whose different experiences and knowledge gave nuanced perspectives during the analysis process.

## Conclusions

Technology can support systematic QI efforts in health care by providing managers and staff with timely and useful feedback on performance. However, the adoption of such technology is a complex process that requires openness to gradual learning and adaptation as well as competent leadership that engages and supports all stakeholders as they seek solutions to the challenges and difficulties that inevitably arise.

## Supplementary information

**Additional file 1:.**

## Data Availability

The datasets generated and/or analysed in this study are not publicly available to maintain confidentially.

## Abbreviations

QI: Quality Improvement
OB/GYN: Obstetrics and Gynecology
NASSS: The Nonadoption, Abandonment, Scale-up, Spread, and Sustainability (NASSS) framework
EMRs: Electronic Health Records
VBHC: Value Based Health Care
