# Supplementary material for: How can technology support quality improvement? Lessons learned from the adoption of an analytics tool for advanced performance measurement in a hospital unit
Source: BMC Health Serv Res. 2020 Sep 1;20:816. doi: 10.1186/s12913-020-05622-7 (PMC7460799; doi:10.1186/s12913-020-05622-7)
Supplement: Supplementary file 1 — Additional file 1:. [file 12913_2020_5622_MOESM1_ESM.docx]

Attachment 1: Interview guide

Focus group interviews with participants in data driven quality improvement in obstetric care

# Participants

Focus group interviews with participants in data driven quality improvement in obstetric care

# Introduction

The purpose of this project is to increase understanding of how health care organizations can improve their ability to increase quality using datadriven QI.

The research project focuses on the ongoing data driven QI initiative in Uppsala. We intend to gain a deeper understanding as to how these initiatives are carried out in practice and identify hindering and supporting factors of the initiatives from your perspective.

The project intends to develop concrete suggestions to develop efficient ways of improving health care.

The duration of the interview will be maximum 90 minutes. The questions focus on the meaning of data driven QI, how you work with data driven QI, how you worked with QI previously, what kind of data you use, what challenges you experience when working this way and what benefits you see.

We have chosen group interviews for two reasons: 1) to take less time from your work with patients and 2) we are interested how you work with QI on a clinic level and not on an individual level. As such, the results will be analyzed on a group level focusing on staff and managers.

The questions are open ended in order for you to discuss your perceptions freely. Participation is voluntary. You can choose to terminate the interview at any time. All data will be treated confidentially, and the recordings will be deleted after transcription. Participation is anonymous and you will not be mentioned by name in any published documents. We will summarize our findings in a research article.

In order to facilitate the analysis, the interview will be recorded. Is that okay with you?

I will now start the recording. And I repeat the question: Is it okay that we record the interview?

## Introductory question

1. Could you describe yourselves shortly (profession, role in the organization, time of employment at this department)?

## QI generally

1. Describe how you work to improve care for your patients?
   1. What does it mean concretely?
   2. Are you using any particular method?
   3. Has your way of working with QI changed over the last years?
   4. Are you focused on any particular group of patients?
   5. What data?
2. **How do you measure and evaluate the care you give?**
   1. What data do you use?

## Technology (data and analytics tool)

As we understand it there is an ongoing activity to follow up your QI work in a systematic and digitalized way at your clinic, with the help of a digital platform.

1. How do you use the tool?
   1. What data used?
   2. How do you use the data?
2. What knowledge/information do you get from the data and the tool?
3. What knowledge is needed to use the data and the tool?
4. What support is needed to use the data and the tool?

## Value proposition and value chain (program theory)

We would like to go back to the time when your clinic started to use the analytics tool.

1. Why did the clinic start to use data and the analytics tool?
   1. With your own words, what problem were you trying to solve?
   2. What were your needs?
   3. What results did you expect?
2. The purpose of Value based Health Care is to measure and evaluate value in health care in order to create the best possible health outcomes for patients. How do you think this purpose has been realized in practice?

## Adopter system

1. How has data and the analytics tool changed your way of working with QI?
   1. Have you used any particular QI model for the project?
   2. How has the managers’ work changed?
   3. How has the staff’s work changed?
   4. What results have you observed with regards to your patients?
      1. Is there and particular patient group or area where you have benefited the most from the data and the analytics tool?
   5. How has your collaboration with other actors (e.g. other clinics at the hospital, other healthcare providers, the county council) changed?
2. What benefits have you experienced compared to your previous way of working with QI?
3. What challenges have you experienced compared to your previous way of working with QI?

## Organization

1. How was the analytics tools introduced?
   1. Who were the key players?
   2. How did you experience the introduction of the tools?
2. What were the conditions for you to work with the analytics tool (e.g. resources, support, time, competence)?

## Wider system

1. How do external factors (e.g. policies or demands from procurement organizations ) affect how you use the data and the analytics tool?

## Embedding and adoption over time

1. What have you learned from the use of the data and the analytics tool?
2. How has the use of the tool developed over time? Have you made any changes?

**Thank you!**
